# Respiratory symptoms up to 18 months after COVID-19 – a longitudinal observational study

**DOI:** 10.1186/s12931-026-03901-1

**Published:** 2026-09-09

**Authors:** Carl Hallberg, Alexandra Halvarsson, Annie Svensson, Anna Törnberg, Anna Svensson-Raskh, Monika Fagevik Olsén, Judith Bruchfeld, Michael Runold, Malin Nygren-Bonnier

**Affiliations:** 1 Division of Physiotherapy, Department of Neurobiology, Care Sciences and Society, Karolinska Institutet, Nobels Allé 23, Huddinge, 141 83 Sweden; 2 Medical Unit Allied Health Professional, Women’s Health and Allied Health Professional Theme, Karolinska University Hospital, Stockholm, Sweden; 3 Department of Health and Rehabilitation/Physiotherapy, Institute of Neuro‑ science and Physiology, Sahlgrenska Academy, University of Gothenburg, Gothenburg, Sweden; 4 Division of Infectious Diseases, Department of Medicine, Solna, Karolinska Institutet and Department of Infectious Diseases, Karolinska University Hospital, Stockholm, Sweden; 5 Division of Respiratory Medicine, Department of Medicine, Solna, Karolinska Institutet and Department of Respiratory Medicine and Allergy, Karolinska University Hospital, Stockholm, Sweden

**Keywords:** Dyspnoea, Lung function, Long COVID, Outcome measures, Physical therapy, Respiratory infection

## Abstract

**Background:**

This study aimed to identify, analyse and compare adults with and without respiratory symptoms over time after COVID-19 and identify predictors of persistent respiratory symptoms, in hospitalised and non-hospitalised patients.

**Methods:**

Participants were recruited at the Post COVID-19 clinic at Karolinska University hospital, Sweden, between May 2020 until December 2022, with longitudinal data collected until February 2025. Data from medical records and two clinical follow-up visits were analysed. Participants were categorised based on presence or absence of self-reported respiratory symptoms. Clinical outcomes included physical function, patient-reported outcomes, lung function and other clinically relevant parameters.

**Results:**

A total of 963 out of 1976 participants were included, of whom 760 (79%) were identified with respiratory symptoms and 203 (21%) were not at first follow-up visit. The respiratory symptom group showed poorer physical function and worse patient-reported outcomes at the first follow-up (adjusted *p* < 0.05). Lung function was generally preserved, although diffusing lung capacity for carbon monoxide (DLCO) was lower in the respiratory symptom group (79% vs 84%, adjusted *p* < 0.05). Both groups improved over time, and the magnitude of change did not differ significantly between groups. However, participants in the respiratory symptom group remained more impaired across several outcomes at follow-up. Presence of respiratory symptoms at the first follow-up visit was the strongest independent predictor of belonging to the respiratory symptom group at second follow-up visit (OR 5.31, 95% CI 2.74–10.89).

**Conclusions:**

Respiratory symptoms after acute COVID-19 were highly prevalent and were associated with poorer clinical outcomes, despite similar recovery over time. Further research is needed to better clarify underlying mechanisms and to guide targeted rehabilitation strategies.

**Supplementary Information:**

The online version contains supplementary material available at https://doi.org/10.1186/s12931-026-03901-1.

## Introduction

A substantial proportion of both hospitalised and non-hospitalised patients with COVID-19 experience persistent symptoms beyond 12 weeks, described as post-COVID-19 condition (PCC) [1]. PCC is a condition affecting multiple organ systems in individuals regardless of sex, age, initial disease severity or prior health status [2]. The World Health Organization estimates that approximately 6% develop PCC after COVID-19 [3]. Population PCC prevalence data from the UK, USA and Canada report rates between 3–6% of which at least 1.5% have severe PCC indicating PCC as an important public health condition [2, 4].

PCC is a multisystem condition, and a wide range of symptoms may thus occur. Common symptoms include fatigue, dyspnoea, dizziness, palpitations, cognitive dysfunction, headache and joint pain [4]. Respiratory symptoms are also frequently reported, with up to approximately 50% of affected individuals experiencing dyspnoea [5]. Other common respiratory symptoms include cough, chest tightness, chest pain and an altered breathing pattern [6–8]. Reported risk factors for developing this respiratory phenotype include obesity, preexisting chronic pulmonary disease, and hospitalization during acute COVID‐19, and it is associated with lower quality of life compared to other PCC-phenotypes [9, 10]. Beyond symptom reports, objective assessments have revealed measurable impairments in lung function [11]. Among the various abnormalities detected with pulmonary function tests, a reduced diffusing capacity of carbon monoxide (DLCO) is the most frequently observed [12].

Longitudinal studies have shown that respiratory symptoms and pulmonary abnormalities can persist over time following acute COVID-19. At 12 months post-infection, between 5–18% of patients previously hospitalised due to COVID-19 reported ongoing respiratory symptoms, while up to 49% exhibit abnormal findings in pulmonary function tests [13, 14]. Longitudinal data indicate that respiratory involvement can persist for years. In one cohort of previously hospitalised patients, 22% still reported respiratory symptoms up to three years after infection, and over one third had persistent radiologic lung abnormalities linked to symptoms and reduced diffusion capacity [11]. In addition to structural and physiological sequelae, dysfunctional breathing may contribute to persistent respiratory symptoms in this population [15].

Despite growing recognition of PCC as a long-term consequence of COVID-19 that can affect both hospitalised and non-hospitalised individuals, important knowledge gaps remain regarding the respiratory phenotype [16]. Most longitudinal studies describe respiratory symptoms and pulmonary abnormalities at group level, but few distinguish between patients with or without respiratory symptoms. Existing studies are often small, restricted to previously hospitalised patients or lack comprehensive physical and patient-reported outcome assessments [11, 13, 14, 17]. Consequently, knowledge remains unclear how self-perceived respiratory symptoms relate to long-term physical outcomes and other standardised patient-reported outcomes assessing health and functioning, and whether these differ between patients with and without such symptoms. Evidence is particularly sparse in mixed cohorts including both hospitalised and non-hospitalised individuals, leaving the respiratory phenotype of PCC insufficiently characterized across the full spectrum of -initial disease severity.

To address these gaps, the aim of this study was to identify, analyse and compare physical function, patient reported outcomes and lung function over time after COVID-19 in a cohort of adult hospitalised and non-hospitalised patients with and without respiratory symptoms. Furthermore, the study aimed to identify predictors of persistent respiratory symptoms.

## Methods

### Design

This longitudinal cohort study is a part of two larger projects called “Recovery and rehabilitation during and after COVID-19” (ReCOV) [18, 19] and “Follow up of severe COVID-19” (Uppcov). Several departments at the Karolinska University Hospital and the Karolinska Institutet collaborate in these projects. Clinical visits were conducted at a specialised, multiprofessional post-COVID outpatient clinic at Karolinska University hospital, Sweden.

### Study population and recruitment

Hospitalised individuals due to COVID-19 were referred to the post-COVID outpatient clinic at discharge if they were treated at intensive care unit (ICU) and/or at a ward requiring more than 2 l/min of oxygen, with extensive pulmonary radiographic changes and/or a complicated clinical course. Non-hospitalised individuals with prolonged symptoms after COVID-19 were referred from primary care or self-referred if they presented symptoms affecting multiple organ systems and persisting for at least 3 months after confirmed or suspected COVID-19 and causing ≥ 50% impaired work ability or equivalent functional impairment.

Participants were recruited while at the clinical visit at the post-COVID-19 clinic between May 2020 and December 2022 and longitudinal data was collected until February 2025. This study is based on data from two standardised clinical visits: The first visit represents the initial follow-up after the acute infection, and the second visit represents the subsequent follow-up assessment. Exclusion criteria for this study included age < 18 years, only digital visit and no available data on self-reported symptoms from first clinical visit.

Participants received oral and written information about the study and were given the opportunity to ask questions about the study and their study participation at the first follow-up visit. The study follows the Helsinki declaration [20] and is approved by the Swedish Ethical Authorities (Dnr 2020–02149, 2020–02394) [19].

### Data collection

Data were collected from patient medical records and included demographic data and clinical data from visits. Demographic variables included age, sex, body mass index (BMI), smoking status, main occupation, sick leave, pre-existing lung disease, comorbidity in postural orthostatic tachycardia syndrome (POTS), time since infection, and hospitalisation course details.

### Clinical outcomes

During clinical visits, data were collected on persistent or newly developed symptoms following COVID-19 illness via face-to-face interviews, physical and lung function tests, and questionnaires about symptoms, physical activity, mental health, and self-rated health. Data were stored and managed using REDCap electronic data capture tools hosted at Karolinska Institutet [21].

### Physical function tests

Physical function was assessed with six-minute walk test (6MWT) and lower extremity strength was measured with one-minute sit-to-stand test (1MSTST). The 6MWT and the 1MSTST were performed according to guidelines and are presented as a percentage of the predicted value [22–25]. Inspiratory muscle strength was assessed using the maximal inspiratory pressure (MIP) test, performed in a standardised manner and in accordance with established guidelines, using a validated respiratory pressure device (Micro-RPM, Care Fusion, San Diego, CA, USA) [26]. The highest pressure was recorded in cm H_2_O and is presented as percentage of the predicted value [27].

### Pulmonary function test

Lung function was measured with dynamic spirometry (Medikro®, Kuopio, Finland) including forced vital capacity (FVC), forced expiratory volume in one second (FEV_1_), peak expiratory flow (PEF) and the FEV_1_/FVC ratio, following ATS/ERS guidelines. All scores were compared to reference values and presented as a percentage of predicted values [28].

Based on clinical indication, a subgroup of participants was referred for extended pulmonary function testing. The extended pulmonary function assessment included dynamic spirometry, static lung volumes measured by body plethysmography and diffusing lung capacity for carbon monoxide (DLCO) through the single breath technique. All scores were compared to reference values and presented as a percentage of predicted values [28].

### Patient-reported outcomes

Overall functional status was assessed using the Post-COVID-19 Functional Status (PCFS) and it consists of five items ranging from 0 (absence of any functional limitation) to 4 (severe functional limitations requiring assistance with activities of daily living) [29]. Participants were instructed to score their functional limitations prior to disease onset and at the visit, the latter covering the last week. Self-reported physical activity was measured with the Frändin-Grimby activity scale, ranging from 1–6, with participants estimating their physical activity level the last two weeks before disease onset and during the last week prior to the actual visit [30]. Dyspnoea severity was assessed using the Modified Medical Research Council dyspnoea scale (mMRC), the scale ranges from 0 to 4, where a score of two or higher indicates clinically relevant dyspnoea [31, 32]. Self-perceived external dyspnoea was registered before and after physical tests (6MWT and 1MSTST) with Borg CR10 (0–10) for dyspnoea [33]. Self-rated health was measured using the EQ-VAS in the EQ-5D instrument, ranging from 0–100 [34, 35]. Mental health was assessed by measuring symptoms of depression and anxiety. Depression was evaluated using the Patient Health Questionnaire‑9 (PHQ‑9), which yields a total score ranging from 0 to 27 [36]. Anxiety was assessed with the Generalised Anxiety Disorder 7‑item scale (GAD‑7), with total scores ranging from 0 to 21 [37]. All patient-reported outcome measures were administered using Swedish versions of questionnaires.

Participants were divided into two groups based on presence or absence of self-reported respiratory symptoms. Respiratory symptoms included dyspnoea, cough, affected breathing pattern, yawning, chest tightness, difficulties or pain when taking deep breaths. Individuals reporting one or more of these symptoms were classified into the respiratory symptom group (RSG), while those reporting none of these symptoms were classified into the non‑respiratory symptom group (Non‑RSG).

### Statistical analyses

Data were summarised using mean (SD), median (IQR), or frequency (n) and proportion (%). To assess between group differences, an independent samples t-test was conducted for continuous variables with normal distribution, while the Mann–Whitney U test was used for those not normally distributed. Categorical variables were analysed using the chi-square test squared or Fisher´s exact test depending on data level. As a sensitivity analysis, the between-group comparisons of clinical outcomes, physical function, lung function, and patient-reported outcomes at the first follow-up visit were repeated after excluding participants classified as Non-RSG who reported clinically relevant dyspnoea (mMRC ≥ 2).

The level of significance was set at *p* < 0.05 for all analyses, and the Benjamini–Hochberg method was used for adjusting for multiple testing [38]. Statistical analyses were conducted using R version 4.5.1 (The R Foundation for Statistical Computing, Vienna, Austria).

### Longitudinal analyses

To examine longitudinal changes and group differences in self-reported symptoms, physical function tests and patient-reported outcomes, linear mixed effect models were applied to compare the RSG and non-RSG. Self-reported symptoms, physical function tests and patient-reported outcomes were analysed in the full cohort. Due to a substantial proportion of participants not completing extended pulmonary function tests at both follow-up visits, analyses were limited to those with data from both visits.

### Regression analyses

To identify factors associated with belonging to the RSG at the second visit, a multivariable logistic regression was performed with RSG (yes/no) at follow-up visit as the dependent variable. Independent variables included age, sex, level of care (hospitalised/non-hospitalised), a patient history of asthma (yes/no), time since initial COVID-19 illness, respiratory symptoms at first follow-up visit (yes/no), and outcome measures at first follow-up visit: 6MWT, MIP-test, 1MSTST, FVC, self-rated health (EQ-VAS), anxiety (GAD-7), depression (PHQ-9), overall functional status (PCFS), physical activity level (Frändin/Grimby), total symptom count (No. of self-reported symptoms), and clinically relevant dyspnoea (mMRC, dichotomised at ≥ 2). Physical and lung function variables were entered into the regression analyses using their original measurement units. Presence of POTS diagnosis (yes/no) (established after COVID-19 illness) was also included. FVC was selected as the lung function variable to avoid multicollinearity among highly correlated pulmonary function measures. Odds ratios with 95% confidence intervals were reported. As a sensitivity analysis, the regression model was repeated without inclusion of respiratory symptom status at the first follow-up visit.

## Results

**Fig. 1:**
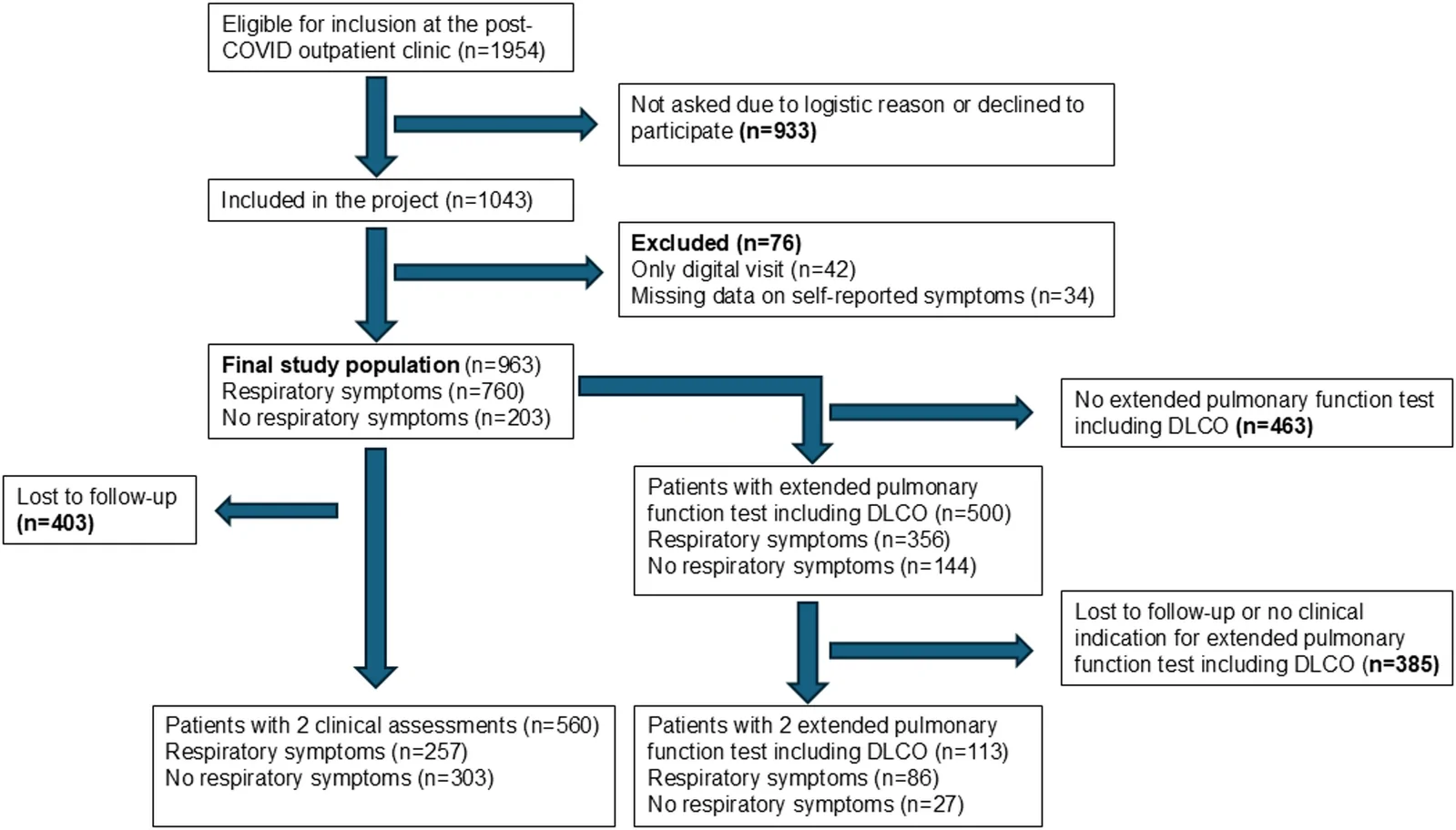
Flowchart of eligible, excluded and included participants

In total, 1976 patients were assessed at the Post COVID outpatient clinic. Of these, 1043 individuals provided consent to participate. After exclusion, due to digital visits only, or missing data of self-reported symptoms, 963 participants remained in the study cohort. Among these, 760 individuals reported respiratory symptoms (RSG) at the first follow-up visit whereas 203 did not (Fig. 1).

In the RSG, 60% were female with a mean age of 50 years. Most participants (53%) had never been hospitalised during their acute infection, compared with 28% in the non-RSG. In the non-RSG, 34% were female with a mean age of 54 years. The two groups were heterogeneous and differed significantly in most demographic and clinical characteristic, except for BMI, pre-existing lung disease, history of asthma, overall health status before COVID-19, days in ICU, and the proportion that needed a mechanical ventilation during hospitalisation. However, they differed significantly in the mean number of days being on mechanical ventilation, where the RSG required a longer duration (9 vs 5 days, adjusted *p* < 0.05) (Table 1).

**Table 1:** Characteristics presented as Demographics, Before COVID-19, and during acute COVID-19 of the total cohort participants, also presented as Non-respiratory symptom group (non-RSG) and Respiratory symptom group (RSG)

	**Total (*n* = 963)**	**non-RSG (*n* = 203)**	**RSG (*n* = 760)**	**Group difference (*p*-value)**
Demographics				
Sex (female)	527 (55%)	69 (34%)	458 (60%)	** < 0.001**
Age	51 (13)	54 (14)	50 (13)	** < 0.001**
BMI, kg/m2	28.0 (6.0)	28.0 (5.8)	28.0 (6.1)	0.957
Missing	3	1	2	
Smoking Status				** < 0.01**
Current smoker	25 (2.7%)	12 (6.2%)	13 (1.8%)	
Former smoker	344 (37%)	65 (34%)	279 (38%)	
Never smoked	554 (60%)	117 (60%)	437 (60%)	
Missing	40	9	31	
Before COVID-19				
Occupational status, pre COVID-19				** < 0.001**
Retired	130 (14%)	45 (23%)	85 (12%)	
Sick leave	33 (3.6%)	2 (1.0%)	31 (4.3%)	
Student	36 (3.9%)	11 (5.5%)	25 (3.4%)	
Work (fulltime or parttime job)	729 (79%)	141 (71%)	588 (81%)	
Missing	35	4	31	
Overall functional status—(PCFS) (pre COVID-19)	0 (0, 0)	0 (0, 0)	0 (0, 0)	0.247
Missing	76	17	59	
Physical activity level—(Frändin/Grimby) (pre COVID-19)	4 (4, 5)	4 (3, 5)	4 (4, 5)	** < 0.05**
Missing	31	10	21	
Lung Disease, pre COVID-19 (Yes)	178 (19%)	30 (15%)	148 (20%)	0.218
Missing	19	6	13	
Asthma, pre COVID-19 (Yes)	161 (17%)	25 (13%)	136 (18%)	0.112
Missing	9	3	6	
During acute COVID-19				
Hospitalized (yes)	502 (52%)	147 (72%)	355 (47%)	** < 0.001**
Hospital Stay (days)	25 (20)	22 (17)	27 (21)	** < 0.01**
ICU Stay (days)	12 (52)	7 (9)	14 (62)	0.056
Ventilator (yes)	213 (44%)	57 (39%)	156 (46%)	0.218
Ventilator (days)	8 (12)	5 (8)	9 (14)	** < 0.001**

Data are presented as n (%); Mean (SD); Median (Q1, Q3). Group differences were assessed using t-test or Mann–Whitney U test for continuous variables and Chi-squared or Fisher´s exact test for categorical variables depending on data level. P-values were corrected for multiple testing using the Benjamini & Hochberg correction for multiple testing, and adjusted p-values are reported

*Abbreviations BMI* Body Mass Index, *ICU* Intensive Care Unit, *PCFS* Post-COVID-19 Functional Status (0–4); *p* < 0.05 considered statistically significant

Median time from initial COVID-19 until the first visit was significantly longer in the RSG compared to the non-RSG (9 vs 5 months, adjusted *p* < 0.05). The two groups differed significantly in most analysed outcomes at first visit (adjusted *p* < 0.05), with generally lower values in the RSG, except for lung function measures (FVC, FEV_1_ and FEV1/FVC ratio), where no significant differences were observed. However, a significant difference was noted in PEF (74% vs 81% adjusted *p* < 0.05) with the RSG demonstrating lower values. The RSG also reported a higher total number of self-reported symptoms (median 8 vs 4), had a larger proportion on 100% sick leave (40% vs 23%), and a higher prevalence of POTS (16% vs 6.9%) compared to the non-RSG (adjusted *p* < 0.05). Significant differences were additionally observed across in all patient-reported outcomes with the RSG being more affected (adjusted *p* < 0.05) (Table 2). In a sensitivity analysis excluding non-RSG participants with clinically relevant dyspnoea (mMRC ≥ 2; *n* = 39), the overall findings remained essentially unchanged (Additional file: Table S1).

**Table 2:** Clinical outcomes, physical function tests, lung function and patient-reported outcomes at first follow-up visit of the total cohort, also presented as Non-respiratory symptom group (non-RSG) and Respiratory symptom group (RSG)

**Clinical outcomes**	**Total** (*n* = 963)	**non-RSG** (*n* = 203)	**RSG** (*n* = 760)	**Group difference (*p*-value)**
Time from acute COVID-19 to first visit (Months)	9 (5, 14); Range: (2, 35)	5 (4, 11); Range: 2, 33	9 (5, 15); Range: 2, 35	** < 0.001**
Missing	16	0	16	
Sick leave				** < 0.001**
0%	244 (35%)	73 (59%)	171 (30%)	
25%	40 (5.7%)	3 (2.4%)	37 (6.0%)	
50%	101 (14%)	11 (8.9%)	90 (16%)	
75%	55 (7.9%)	9 (7.3%)	46 (8.0%)	
100%	257 (37%)	28 (23%)	229 (40%)	
Missing	266	79	187	
POTS				** < 0.001**
No POTS	824 (86%)	189 (93%)	635 (84%)	
POTS	139 (14%)	14 (6.9%)	125 (16%)	
Total symptoms (No. of self-reported symptoms)	7 (4, 11); Range: 0, 30	4 (1, 6); Range: 0, 17	8 (4, 11); Range: 0, 30	** < 0.001**
Physical function tests				
6MWT (% of predicted)	82 (24)	92 (20)	80 (24)	** < 0.001**
Missing	30	8	22	
MIP (% of predicted)	89 (30)	95 (29)	87 (30)	** < 0.001**
Missing	22	8	14	
1MSTST (% of predicted)	64 (26)	72 (27)	62 (26)	** < 0.001**
Missing	64	8	56	
Pulmonary function test (dynamic spirometry)				
FVC (% of predicted)	81 (16)	82 (16)	80 (16)	0.348
Missing	104	21	83	
FEV_1_ (% of predicted)	82 (16)	83 (16)	81 (16)	0.139
Missing	104	21	83	
FEV_1_/FVC ratio	0.79 (0.08)	0.79 (0.08)	0.79 (0.08)	0.918
Missing	85	16	69	
PEF (% of predicted)	75 (19)	81 (20)	74 (19)	** < 0.001**
Missing	111	23	88	
Patient-reported outcomes				
Self-rated health (EQ-VAS)	52 (22)	64 (23)	48 (21)	** < 0.001**
Missing	128	34	94	
Self-perceived external dyspnoea at rest (Borg CR10)	0.5 (0, 2); Range: 0, 8	0 (0, 0); Range: 0, 5	1 (0, 2.50); Range: 0, 8	** < 0.001**
Missing	34	8	26	
Self-perceived external dyspnoea after 1MSTS (Borg CR10)	5 (3, 7); Range: 0, 11	3 (3, 5); Range: 0, 9	5 (3, 7); Range: 0, 11	** < 0.001**
Missing	171	48	123	
Self-perceived external dyspnoea after 6MWT (Borg CR10)	4 (3, 6); Range: 0, 11	3 (2, 4); Range: 0, 9	4 (3, 7); Range: 0, 11	** < 0.001**
Missing	57	12	45	
Dyspnoea severity (mMRC) Clinically relevant dyspnoea (mMRC ≥ 2)	2 (1, 3); Range: 0, 4 468 (55%)	1 (0.50, 1); Range: 0, 4 39 (22%)	2 (1, 3); Range: 0, 4 429 (64%)	** < 0.001** ** < 0.001**
Missing	113	27	86	
Overall functional status (PCFS)	2 (1, 3); Range: 0, 4	0 (0, 2); Range: 0, 4	3 (1, 3); Range: 0, 4	** < 0.001**
Missing	53	10	43	
Physical activity level (Frändin/Grimby)	3 (2, 3); Range: 1, 6	3 (3, 4); Range: 1, 6	3 (2, 3); Range: 1, 6	** < 0.001**
Missing	27	10	17	
Depression (PHQ-9)	9 (4, 13); Range: 0, 27	5 (2, 9); Range: 0, 23	9 (5, 14); Range: 0, 27	** < 0.001**
Missing	98	17	81	
Anxiety (GAD-7)	4 (1, 9); Range: 0, 21	3 (0, 7); Range: 0, 19	5 (2, 10); Range: 0, 21	** < 0.001**
Missing	89	15	74	

Data are presented as n (%); Mean (SD); Median (Q1, Q3); Range: Min, Max. Group differences were assessed using t-test or the Wilcoxon rank-sum test for continuous variables and Chi-squared or Fisher´s exact test for categorical variables depending on data level. *P*-values were corrected for multiple testing using the Benjamini & Hochberg correction for multiple testing, and adjusted *p*-values are reported

*Abbreviations* EQ-VAS (0–100)—in the EuroQol Visual Analogue Scale, *FEV*_*1*_ Forced Expiratory Volume in 1 s, *FEV*_*1*_*/FVC* Ratio of FEV_1_ to FVC, *FVC* Forced Vital Capacity, *GAD-7* Generalized Anxiety Disorder-7 (0–21), *mMRC* Modified Medical Research Council dyspnea scale (0–4), *MIP* Maximal Inspiratory Pressure, *PCFS* Post-COVID-19 Functional Status (0–4), *PEF* Peak Expiratory Flow, *PHQ-9* Patient Health Questionnaire-9 (0–27), *POTS* Postural Orthostatic Tachycardia Syndrome, *1MSTST* 1-Minute Sit-to-Stand Test, *6MWT* 6-Minute Walk Test; *p* < 0.05 considered statistically significant

**Fig. 2:**
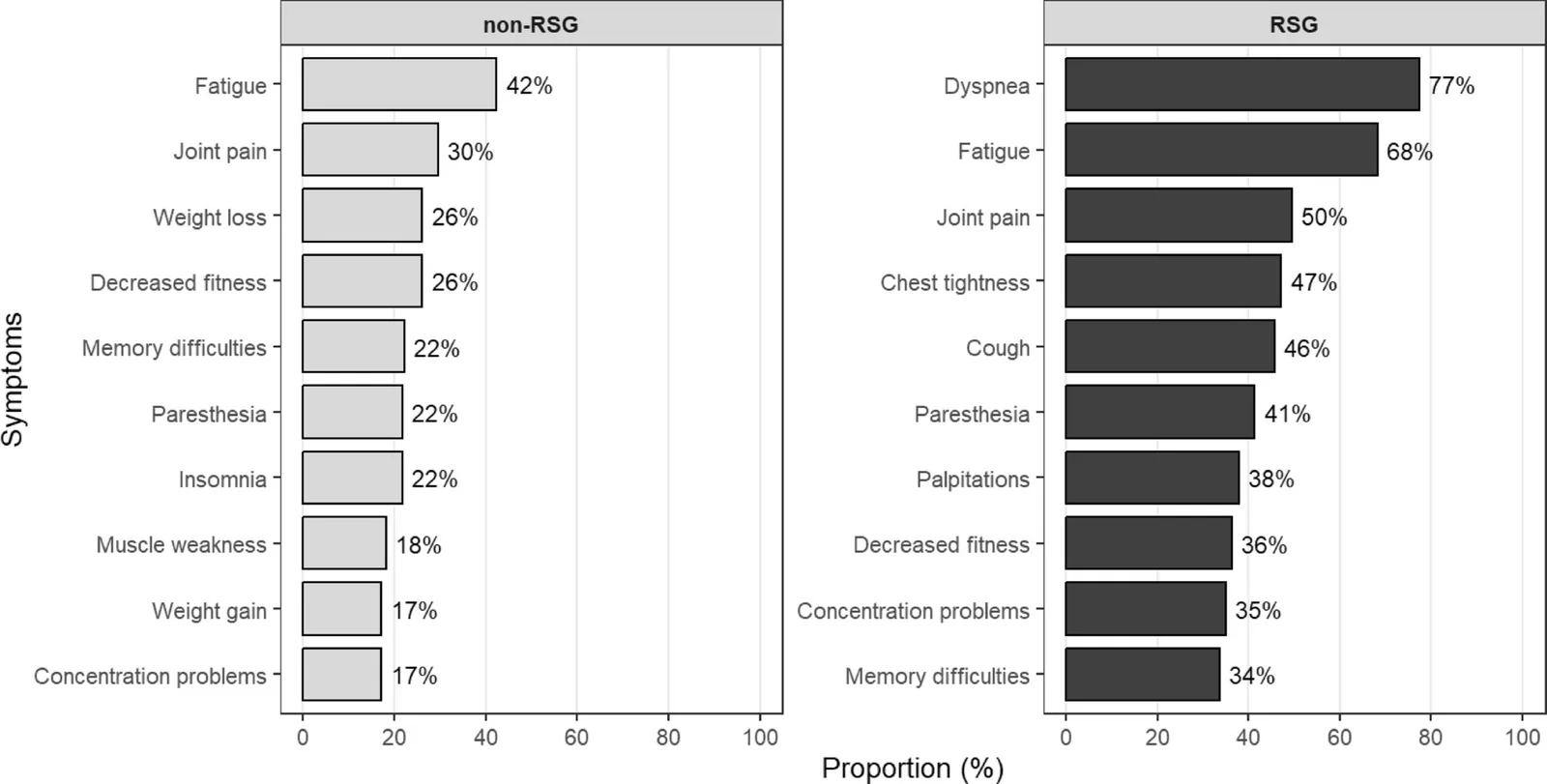
Ten most frequently self-reported symptoms at the first follow‑up visit, presented as proportions (%) and stratified by groups (Non‑respiratory symptom group (non-RSG) (*n* = 203) and Respiratory symptom group (RSG) (*n* = 760))

The most common self-reported symptom in the RSG was dyspnoea followed by fatigue and joint pain. In the non-RSG, fatigue was the most frequently reported self-reported symptom followed by joint pain and weight loss. Notably, although fatigue was frequently reported in both groups, it was substantially more common in the RSG (68% vs. 42%). Overall, the RSG reported higher symptom prevalence across most symptom categories, whereas the non-RSG displayed lower and evenly distributed prevalence levels (Fig. 2).

Pulmonary function testing was available for a subgroup of participants (*n* = 500) at the first visit. All measured lung function parameters were within normal ranges for both groups. The only significant difference was observed for DLCO, which was lower in the RSG compared to the non-RSG (79% vs 84%, adjusted *p* < 0.05). A significant larger proportion of participants in the RSG also had a DLCO value below 80% of predicted (50% vs 33%, adjusted *p* < 0.05) (Table 3).

**Table 3:** Extended pulmonary function test results for the total cohort at first visit, presented as precent of predicted value and proportion with < 80% of predicted value, also presented as Non-respiratory symptom group (non-RSG) and Respiratory symptom group (RSG)

	**Total** (*n* = 500)	**non-RSG** (*n* = 144)	**RSG** (*n* = 356)	**Group difference** **(*p*-value)**
Time to PFT (Months)	10 (8, 15)	10 (8, 11)	11 (9, 17)	** < 0.001**
FVC (% of predicted) FVC < 80% predicted	87 (17) 138 (28%)	89 (17) 38 (26%)	87 (17) 100 (28%)	0.413 0.857
FEV_1_ (% of predicted) FEV_1_ < 80% predicted	88 (17) 146 (29%)	90 (16) 36 (25%)	87 (17) 110 (31%)	0.413 0.584
FEV_1_/FVC ratio	0.78 (0.08)	0.78 (0.08)	0.79 (0.08)	0.776
VC (% of predicted) VC < 80% predicted	87 (16) 156 (31%)	89 (16) 39 (27%)	86 (16) 117 (33%)	0.413 0.584
TLC (% of predicted) TLC < 80% predicted	90 (16) 119 (24%)	91 (15) 33 (23%)	90 (16) 86 (25%)	0.925 0.857
FRC (% of predicted) FRC < 80% predicted	105 (89) 100 (20%)	113 (156) 32 (22%)	102 (37) 68 (19%)	0.530 0.857
RV (% of predicted) RV < 80% predicted	104 (35) 106 (21%)	101 (38) 32 (22%)	105 (34) 74 (21%)	0.530 0.857
DLCO (% of predicted) DLCO < 80% predicted	81 (18) 226 (45%)	84 (17) 48 (33%)	79 (18) 178 (50%)	** < 0.05** ** < 0.01**

Data are presented as n (%); Mean (SD); Median (Q1, Q3); Group differences were assessed using t-test or the Wilcoxon rank-sum test for continuous variables and Chi-squared or Fisher´s exact test for categorical variables depending on data level. *P*-values were corrected for multiple testing using the Benjamini & Hochberg correction for multiple testing, and adjusted p-values are reported

*Abbreviations DLCO* Diffusing Capacity for Carbon Monoxide, *FEV*_*1*_ Forced Expiratory Volume in 1 s, *FEV*_*1*_*/FVC* Ratio of FEV_1_ to FVC, *FRC* Functional Residual Capacity, *FVC* Forced Vital Capacity, *PFT* Pulmonary function test, *RV* Residual Volume, *TLC* Total Lung Capacity, *VC* Vital Capacity; *p* < 0.05 considered statistically significant

### Longitudinal changes in physical function tests and patient-reported outcomes

**Fig. 3:**
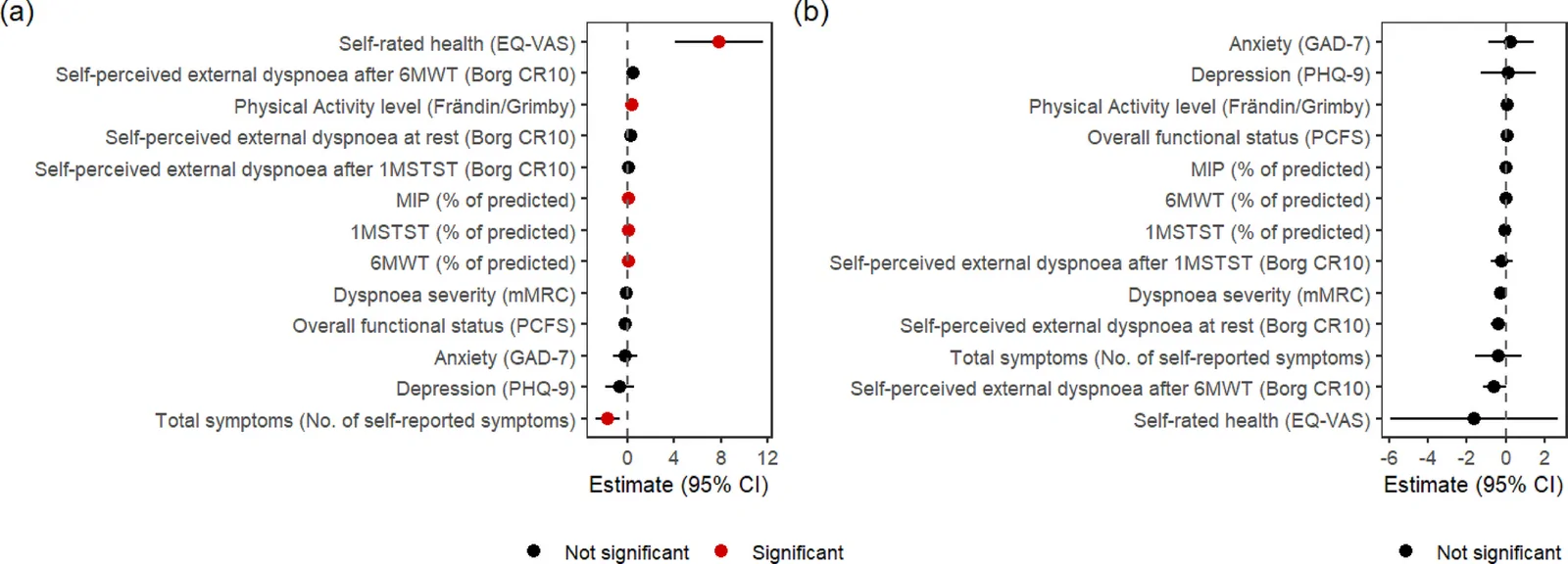
Forest plots showing fixed-effect estimates with 95% confidence intervals from the linear mixed-effects models. The estimates represent unstandardized model coefficients expressed in the original units of each outcome measure. Panel (**a**) shows the main effect of time, representing the change from T1 to T2 across both groups. Panel (**b**) shows the time × group interaction, representing the difference in change from T1 to T2 between the Non-respiratory symptom group (non-RSG) and the Respiratory symptom group (RSG)

Median time from acute COVID-19 to second follow-up visit was 18 months overall, and significantly longer in the RSG compared to the non-RSG (19 vs 12 months, adjusted *p* < 0.05). Changes over time did not differ significantly between groups for any of the outcomes (adjusted *p* > 0.05) (Fig. 3b). Both groups improved over time in physical function (6MWT; Additional file: Fig. S1a, 1MSTST), inspiratory muscle strength (MIP), self-reported physical activity (Frändin/Grimby), self-rated health (EQ-VAS; Additional file: Fig. S1b), and symptom burden (adjusted *p* < 0.05 for time effect), while mental health (PHQ-9; Additional file: Fig. S1c, GAD-7) and PCFS remained largely unchanged. Dyspnoea measures, including mMRC and Borg CR10 (at rest and post-test), did not change significantly over time, and the RSG consistently reported higher scores (adjusted *p* < 0.05) (Fig. 3a). Between-group differences at first follow-up visit persisted for most outcomes, with the RSG showing lower exercise capacity, inspiratory muscle strength, and self-rated health, as well as higher symptom burden and dyspnoea scores (all adjusted *p* < 0.05) at second follow-up visit (Additional file: Table S2).

### Longitudinal changes in lung function

**Fig. 4:**
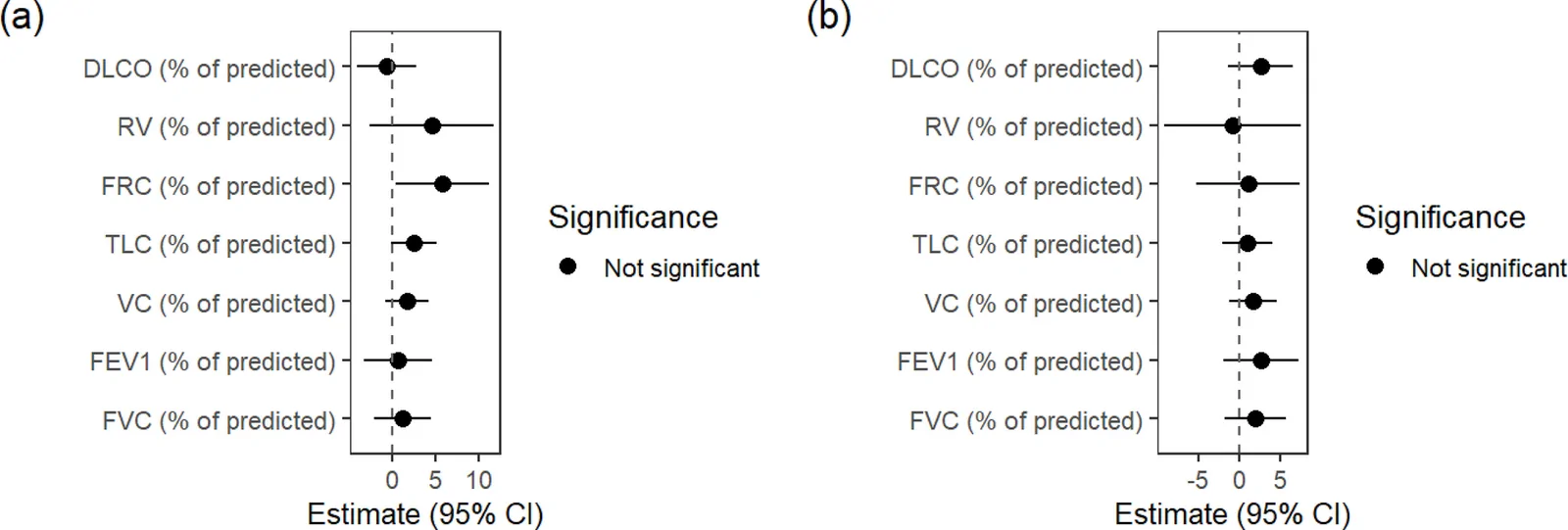
Forest plots showing fixed-effect estimates with 95% confidence intervals from linear mixed-effects models for extended pulmonary function tests. The estimates represent unstandardized model coefficients expressed in the original units of each outcome measure. Panel (**a**) shows the main effect of time, representing the change from the first follow-up visit (T1) to the second follow-up visit (T2) across both groups. Panel (**b**) shows the time × group interaction, representing the difference in change from T1 to T2 between the Non-respiratory symptom group (non-RSG) (*n* = 27) and the Respiratory symptom group (RSG) (*n* = 86)

Longitudinal analyses were conducted on participants with extended pulmonary function test data at both follow‑up visits (*n* = 113). Changes in lung function over time did not differ significantly between groups for any parameter (all adjusted *p* > 0.05; Fig. 4b), and no significant changes over time were observed in the full cohort (Fig. 4a). Within-group changes were generally modest. Individuals in the RSG showed significant improvements in several lung function measures over time, whereas changes in the non-RSG were smaller and mostly not significant (Additional file: Table S3 and Fig. S1d).

### Factors associated with respiratory symptoms at second follow-up visit

Of the 560 individuals who attended the second follow-up visit, 257 (46%) reported respiratory symptoms and 303 (54%) reported no respiratory symptoms (Additional file: Fig. S2). The percentage distribution of specific respiratory symptoms at both follow‑up visits, illustrated which symptoms persisted or declined over time. While dyspnoea decreased over time, other respiratory symptoms, including cough, chest tightness, altered breathing pattern, and difficulties with deep breathing, remained prevalent at the second follow-up visit (Additional file: Fig. S3). Several variables showed significant associations in univariable analyses. However, in the multivariable logistic regression, the presence of respiratory symptoms at the first follow-up visit was the strongest independent predictor of belonging to the RSG at second follow-up visit (OR 5.31, 95% CI 2.74–10.89, adjusted p < 0.05). Clinically relevant dyspnoea (mMRC ≥ 2) showed a nominal association and lower exercise capacity (6MWT) remained associated before correction for multiple testing, but neither variable remained significant after adjustment. No other variables were independently associated with respiratory symptom status at follow-up (Table 4). The results were consistent with those obtained in the sensitivity analysis excluding respiratory symptom status at the first follow-up visit from the model (Additional file: Table S4).

**Table 4:** Univariable and multivariable logistic regression analyses (*n* = 560) for predictors of belonging to the Respiratory symptom group (RSG) at the second follow-up visit (median 18 months post infection)

	**Univariat**					**Multivariat**			
	**n**	**OR**	**95% CI**	***p*-value**	**p.adj (BH)**	**OR**	**95% CI**	***p*-value**	**p.adj (BH)**
First visit assessment									
6MWT (meter)	545	1.00	1.00, 1.00	** < 0.001**	** < 0.01**	1.00	1.00, 1.00	** < 0.05**	0.291
MIP (cmH_2_O)	549	1.00	0.99, 1.00	0.061	0.086	1.00	0.99, 1.01	0.920	0.974
1MSTST (No. of repetitions)	541	0.97	0.95, 0.99	** < 0.01**	** < 0.01**	1.02	0.98, 1.05	0.315	0.847
Self-rated health (EQ-VAS)	504	0.99	0.98, 1.00	** < 0.01**	** < 0.05**	1.01	1.00, 1.03	0.193	0.847
Depression (GAD-7)	520	1.05	1.02, 1.08	** < 0.01**	** < 0.05**	1.01	0.95, 1.07	0.856	0.963
Anxiety (PHQ-9)	527	1.05	1.01, 1.08	** < 0.01**	** < 0.01**	1.00	0.94, 1.06	0.974	0.974
Overall functional status (PCFS)	538	1.24	1.09, 1.42	** < 0.01**	** < 0.01**	1.09	0.84, 1.40	0.511	0.847
Physical Activity level (Frändin/Grimby)	546	0.76	0.64, 0.90	** < 0.01**	** < 0.01**	1.15	0.84, 1.57	0.383	0.847
Total symptoms (No. of self-reported symptoms)	560	1.07	1.03, 1.11	** < 0.001**	** < 0.01**	1.03	0.96, 1.10	0.449	0.847
FVC (L)	523	0.76	0.63, 0.92	** < 0.01**	** < 0.05**	0.85	0.61, 1.18	0.337	0.847
Respiratory symptoms (Yes)	560	5.03	3.11, 8.47	** < 0.001**	** < 0.001**	5.31	2.61, 11.5	** < 0.001**	** < 0.001**
Clinically relevant dyspnoea (mMRC ≥ 2) (Yes)	486	5.71	2.37, 17.0	** < 0.001**	** < 0.01**	4.47	1.39, 17.7	** < 0.05**	0.097
Time from acute COVID-19 to first visit (Months)	554	1.00	0.98, 1.03	0.735	0.826	1.01	0.97, 1.07	0.574	0.847
Sex (F)	560	1.47	1.05, 2.06	** < 0.05**	** < 0.05**	0.93	0.48, 1.81	0.830	0.963
Age	560	1.00	0.99, 1.01	0.948	0.976	1.00	0.97, 1.02	0.719	0.925
POTS (Yes)	560	1.01	0.62, 1.64	0.976	0.976	0.83	0.40, 1.70	0.612	0.847
Hospitalized (Yes)	560	0.89	0.64, 1.24	0.492	0.590	1.43	0.60, 3.46	0.425	0.847
Asthma (Yes)	555	1.24	0.79, 1.93	0.350	0.450	0.83	0.44, 1.56	0.743	0.810

*P*-values were corrected for multiple testing using the Benjamini & Hochberg correction for multiple testing, and adjusted *p*-values are reported, Time to second follow-up visit, median 18 months

*Abbreviations CI* Confidence Interval, *GAD-7* Generalized Anxiety Disorder-7 (0–21), *mMRC* Modified Medical Research Council dyspnea scale using ≥ 2 as a cut off, *MIP* Maximal Inspiratory Pressure, *OR* Odds ratio, *PCFS* Post-COVID-19 Functional Status (0–4), *PHQ-9* Patient Health Questionnaire-9 (0–27), *POTS* Postural Orthostatic Tachycardia Syndrome, *EQ-VAS (0–100)*- in the EuroQol Visual Analogue Scale, *1MSTST* 1-Minute Sit-to-Stand Test, *6MWT* 6-Minute Walk Test, *p* < 0.05 considered statistically significant

## Discussion

In this longitudinal observational follow-up study of individuals with previous COVID-19, 79% were identified with respiratory symptoms at 9 months and 46% at 18 months after acute COVID-19. Improvements over time in clinical outcomes including physical function, inspiratory muscle strength, self-rated health and symptom burden were seen in all participants. However, individuals with respiratory symptoms consistently exhibited poorer outcomes across all analysed measures. Moreover, clinically relevant dyspnoea, with mMRC score of 2 or above at the 9 months was an independent predictor of belonging to the RSG at 18 months.

The high prevalence of respiratory symptoms in our cohort (79%) aligns with previous research suggesting that dyspnoea is one of the most common persistent symptoms of PCC [5]. Participants in the RSG were predominantly female, younger, and non-hospitalised during acute COVID-19 infection, compared to participants in the non-RSG. Importantly, our study cohort is a clinically selected cohort referred to a specialist post-COVID-19 clinic. It includes both previously hospitalised individuals with severe pneumonia, acute respiratory distress syndrome (ARDS), prolonged hospitalisation and mechanical ventilation, and non-hospitalised individuals with severe PCC leading to reduced work ability or comparable functional impairment. This context supports the understanding that PCC is a multisystem condition that can affect individuals regardless of the severity of their initial COVID-19 [2]. Interestingly, a larger proportion of participants in the non-RSG had been hospitalised during the acute infection. This may partly reflect the composition of the study cohort and the different referral pathways to the specialised post-COVID outpatient clinic. Furthermore, hospitalisation was not independently associated with respiratory symptom status at follow-up in the multivariable analysis. The observed differences between groups may also reflect the heterogeneous nature of PCC and potentially distinct clinical manifestations or phenotypes within this population.

At the first follow-up, individuals in the RSG demonstrated significantly worse results across all clinical outcomes, except for lung function measures (FVC, FEV_1_ and FEV_1_/FVC ratio). The observation of high proportions of sick leave in the RSG indicates that respiratory symptoms have a substantial impact on work ability. These findings are consistent with the respiratory PCC phenotype and its association with reduced quality of life [10]. Taken together, these findings suggest that respiratory symptoms may reflect more than pulmonary dysfunction and may instead represent a broader burden of disease in PCC. Individuals with respiratory symptoms consistently demonstrated worse physical function, greater symptom burden, reduced work ability, and poorer patient-reported outcomes despite largely preserved lung function measures. This supports the view that respiratory symptoms may represent one component of a broader multisystem PCC phenotype. The present study extends previous longitudinal PCC research by demonstrating that respiratory symptoms identify a subgroup of individuals with consistently greater functional impairment and symptom burden over time. Although several longitudinal studies have reported longer follow-up periods and incorporated advanced pulmonary imaging [11, 17], our findings suggest that respiratory symptoms provide clinically relevant information beyond standard lung function measures and may help identify individuals at risk of a more burdensome and prolonged recovery.

Although physical function and self-rated health improved over time in both groups, individuals in the RSG remained more impaired at the second follow-up. This persistent gap indicates that individuals with early respiratory symptoms have a more protracted and incomplete recovery. This interpretation aligns with other longitudinal studies indicating a non-linear recovery and may plateau over time [39–41]. The difference in follow-up timing between groups should, however, be considered when interpreting these findings. Despite a longer time since infection, participants in the RSG remained more impaired across most outcomes, and time from acute COVID-19 to the first follow-up visit was not associated with respiratory symptom status at follow-up in the regression analyses. Together, these findings underscore the need for a longer follow-up of this patient group.

A key finding was the discrepancy between subjective respiratory symptoms and objective lung function measurements. FVC, FEV_1_ did not differ between groups, whereas PEF and DLCO was significantly lower in the RSG. Since PEF is highly effort‑dependent and sensitive to suboptimal technique, measurement variability should also be considered when interpreting this finding [42]. The discrepancy suggests that while overall lung volumes are largely preserved, impaired gas exchange may be a physiological marker for persistent respiratory symptoms. This aligns with recent findings where reduced DLCO was a strong independent predictor of reduced physical capacity and persistent breathlessness up to three years after initial infection [39]. The absence of measurable improvements may reflect that lung function was already within normal limits at first follow-up visit. However, since pre-COVID-19 baseline lung function was not available, the possibility that some individuals had higher values then average prior to the infection cannot be excluded.

Several mechanisms can be considered to explain the reduced DLCO. Functional respiratory imaging by Svensson et al. (2025) has demonstrated long-term vascular remodelling in previously hospitalised individuals with PCC, including reduced perfusion of small peripheral vessels, indicating persistent microvascular injury, likely driven by endothelial and immune mediated damage [43]. Furthermore, structural changes in the small airways appear to contribute to the reduced DLCO as bronchial dilatation has been strongly associated with impaired diffusion, suggesting that airway remodelling plays an important role alongside vascular injury [44]. Although prior studies of hospitalised patients with PCC report improvements in lung function over time, most measures remain within normal ranges [11, 13]. Therefore, the gains in physical function and patient-reported outcomes in our cohort may reflect extrapulmonary mechanisms, although pulmonary changes not captured by the lung function measures included in this study cannot be excluded.

The presence of respiratory symptoms at the first follow-up visit was the strongest predictor of respiratory symptoms at the second follow-up visit (OR 5.31). This suggests that individuals who continue to experience respiratory symptoms within the first months after COVID-19 are at increased risk of long-term symptom persistence. Clinically relevant dyspnoea (mMRC ≥ 2) also showed an association with respiratory symptoms at follow-up, although this did not remain significant after correction for multiple testing. Nevertheless, dyspnoea remains clinically relevant, as previous studies have identified persistent dyspnoea as a marker of prolonged recovery after COVID-19 [39, 45]. Previously reported from our research group, clinically relevant dyspnoea was also associated with lower self-rated health in a mixed cohort of hospitalised and non-hospitalised individuals with PCC [18]. From a clinical perspective, early identification of patients with persistent respiratory symptoms and dyspnoea may therefore be important for clinical management and rehabilitation.

Notably, dyspnoea was also the most frequently self-reported symptom within the RSG at the first follow‑up, which supports the construct validity of our grouping. Given that mMRC is part of the international Core Outcome Measurement Set (COMS) for PCC, its use in our study represents a methodological strength and facilitates comparability across cohorts [46]. The choice to use mMRC as an outcome rather than a grouping variable was due to missing data at first follow-up. This approach maximised analysable data while still demonstrating the prognostic value. The high odds ratio observed suggests that mMRC is not merely descriptive but also an indicator of long-term recovery trajectories.

Breathing related symptoms, however, encompass more than dyspnoea severity alone. Classifying patients using a broader set of self-reported respiratory symptoms such as cough, chest tightness, affected breathing pattern, difficulties with deep breathing and yawning captures dimensions of breathing difficulties that might not fully be reflected by dyspnoea severity alone. These symptoms are often characteristic of dysfunctional breathing, a condition that can persist despite pulmonary function tests [15, 47]. This suggests that the respiratory phenotype of PCC involves not only structural or vascular factors but also altered sensation and control. Persistent chest tightness or an increased breathing effort likely contributes to the markedly lower self-rated health observed in this group. When the most basic act of breathing is compromised, the perceived overall disease burden becomes substantial [10]. However, the inclusion of a broad range of respiratory symptoms was intended to capture the wider respiratory phenotype of PCC and does not imply a common underlying pathophysiology. Consequently, the respiratory symptom group should be regarded as a heterogeneous clinical group. Future studies should investigate whether specific respiratory symptom profiles are associated with distinct clinical characteristics and long-term outcomes.

The strength of this study is the longitudinal design with two follow-up visits, allowing evaluation of changes over time and long-term outcomes. Another strength is the use of both objective and subjective outcome measures that are used in clinical care as well as in research, which increases the clinical relevance and applicability of the results. The study also benefits from a large patient population which in addition includes both hospitalised and non-hospitalised individuals.

### Limitations

The findings of this study should be interpreted in the context of its limitations. Due to the longitudinal observational design, only identification of associations and trends over time can be identified but not casual inference. The cohort was recruited from a single specialist post-COVID-19 clinic and consisted of individuals with more advanced PCC, which limits the external validity and generalisability to less severe PCC. Approximately half of the eligible patients participated in both the ReCOV and Uppcov projects. Variations in referral pathways, recruitment procedures, logistics, and clinical routines meant that some individuals were not approached. This likely introduced selection bias and limited generalisability. Group classification relied on self-reported respiratory symptoms data. As described, many symptoms can persist following a COVID-19 illness and individuals may prioritize certain complaints leading to under- or over-reporting of some symptoms. Differences in wording and in how it is interpreted by healthcare professionals can also lead to variations in symptom reporting. These factors likely contributed to the observation that some participants assigned to the non‑respiratory group nonetheless had mMRC ≥ 2, reflecting a mismatch between symptom occurrence as reported in the clinical interview and dyspnoea severity captured by mMRC dyspnoea scale. Although symptom transitions between follow-up visits were described, factors associated with resolution or development of respiratory symptoms were not specifically investigated. Future studies should address these transitions to improve understanding of recovery trajectories in PCC. The inclusion of both hospitalised and non-hospitalised patients also presents challenges, as they had different follow-up routines, due to the lack of knowledge and available resources for the non-hospitalised group at the time of the study. These factors contributed to missing data and reduced sample size for some variables. Participants were recruited between May 2020 and December 2022, during a period when SARS-CoV-2 variants, vaccination coverage, clinical management, and reinfection rates changed substantially. These factors may have influenced symptom burden, clinical presentation, and recovery. However, detailed and complete data regarding viral variants, vaccination status, and reinfections were not consistently available and could therefore not be accounted for in the analyses. The fact that data collection took place during an ongoing pandemic also contributed to missing data, as optimal resources were not always available as well as participants were unable to attend visits due to restrictions and resource constraints.

## Conclusion

In conclusion, respiratory symptoms following a COVID-19 were highly prevalent in this study cohort. Patients with respiratory symptoms demonstrated lower physical function and reported worse patient-reported outcomes that persisted over time, although their recovery appeared similar to those without respiratory symptoms. These findings highlight the substantial functional impact of respiratory symptoms and underscore the importance of early identification of patients at risk for long-term limitations.

Further studies should investigate whether these differences remain beyond the current follow-up period. They should also explore the underlying mechanisms behind persistent respiratory symptoms despite largely preserved lung function. Interventional research is also needed to determine which rehabilitation strategies can optimize recovery in this group of patients.

## Supplementary Information

Additional file 1.

## Data Availability

Data is not publicly available, but available upon request. Requests for access to the data can be put to our Research Data Office (rdo@ki.se) at Karolinska Institutet and will be handled according to the relevant legislation. This will require a data processing agreement or similar with the recipient of the data.

## Abbreviations

1MSTST: 1-Minute sit-to-stand test
6MWT: Six-minute Walk Test
BMI: Body mass index
DLCO: Diffusing Capacity for Carbon Monoxide
EQ VAS: EuroQol visual analogue scale
FEV_1_: Forced expiratory volume in one second
FEV_1_/FVC: Ratio of FEV1 to FVC
FRC: Functional Residual Capacity
Frändin/Grimby: Frändin/Grimby activity score
FVC: Forced vital capacity
GAD-7: General Anxiety Disorder 7-item scale
ICU: Intensive Care Unit
MIP: Maximal inspiratory pressure
mMRC: Modified Medical Research Council Dyspnea Scale
PFT: Pulmonary function test
PHQ-9: Patient Health Questionnaire-9
PCC: Post-COVID-19 condition
POTS: Postural orthostatic tachycardia syndrome
ReCOV: Recovery and rehabilitation during and after COVID-19
RV: Residual Volume
TLC: Total Lung Capacity
Uppcov: Follow up of severe COVID-19
VC: Vital Capacity
