# Supplementary material for: Respiratory symptoms up to 18 months after COVID-19 – a longitudinal observational study
Source: Respir Res. 2026 Sep 9;27:347. doi: 10.1186/s12931-026-03901-1 (PMC13560428; doi:10.1186/s12931-026-03901-1)
Supplement: Supplementary file 1 [file 12931_2026_3901_MOESM1_ESM.pdf]

Supplementary material

Table S1. Sensitivity analysis of clinical outcomes, physical function tests, lung function and patient-reported outcomes at first follow-up visit of the total cohort, Non-respiratory symptom group (Non-RSG), and Respiratory symptom group (RSG), after exclusion of Non-RSG participants with clinically relevant dyspnoea (mMRC ≥2).

**Clinical outcomes**	**Total** (n = 924)	**non-RSG** (n = 164)	**RSG** (n = 760)	**Group difference (p-value)**
**Time from acute COVID-19 to first visit (Months)**	9 (5, 14); Range: (2, 35)	5 (4, 10); Range: 2, 33	9 (5, 15); Range: 2, 35	**<0.001**
Missing	16	0	16	
**Sick leave**				**<0.001**
0%	239 (36%)	68 (69%)	171 (30%)	
25%	39 (5.8%)	2 (2.0%)	37 (6.0%)	
50%	98 (15%)	8 (8.1%)	90 (16%)	
75%	51 (7.6%)	5 (5.1%)	46 (8.0%)	
100%	245 (36%)	16 (16%)	229 (40%)	
Missing	252	65	187	
**POTS**				**<0.001**
No POTS	794 (86%)	159 (97%)	635 (84%)	
POTS	130 (14%)	5 (3.0%)	125 (16%)	
**Total symptoms** (No. of self-reported symptoms)	7 (4, 11); Range: 0, 30	3 (1, 6); Range: 0, 17	8 (4, 11); Range: 0, 30	**<0.001**
**Physical function tests**				
**6MWT (% of predicted)**	82 (24)	96 (18)	80 (24)	**<0.001**
Missing	28	6	22	
**MIP (% of predicted)**	89 (30)	97 (29)	87 (30)	**<0.001**
Missing	21	7	14	
**1MSTST (% of predicted)**	64 (26)	74 (27)	62 (26)	**<0.001**
Missing	61	5	56	
**Pulmonary function test (dynamic spirometry)**				
**FVC (% of predicted)**	81 (16)	82 (16)	80 (16)	0.255
Missing	100	17	83	
**FEV_1 (% of predicted)**	82 (16)	84 (16)	81 (16)	0.061
Missing	100	17	83	
**FEV_1/FVC ratio**	0.79 (0.08)	0.79 (0.08)	0.79 (0.08)	0.892
Missing	82	13	69	
**PEF (% of predicted)**	75 (19)	82 (21)	74 (19)	**<0.001**
Missing	107	19	88	
**Patient-reported outcomes**				
**Self-rated health (EQ-VAS)**	52 (22)	70 (19)	48 (21)	**<0.001**
Missing	126	32	94	
**Self-perceived external dyspnoea at rest (Borg CR10)**	0.5 (0, 2); Range: 0, 8	0 (0, 0); Range: 0, 5	1 (0, 2.50); Range: 0, 8	**<0.001**
Missing	33	7	26	
**Self-perceived external dyspnoea after 1MSTS (Borg CR10)**	5 (3, 7); Range: 0, 11	3 (3, 5); Range: 0, 8	5 (3, 7); Range: 0, 11	**<0.001**
Missing	161	38	123	
**Self-perceived external dyspnoea after 6MWT (Borg CR10)**	4 (3, 6); Range: 0, 11	3 (2, 4); Range: 0, 9	4 (3, 7); Range: 0, 11	**<0.001**
Missing	54	9	45	
**Dyspnoea severity (mMRC)**<br>**Clinically relevant dyspnoea (mMRC ≥ 2)**	2 (1, 3); Range: 0, 4<br>429 (53%)	1 (0, 1); Range: 0, 1<br>0 (0%)	2 (1, 3); Range: 0, 4<br>429 (64%)	**<0.001**<br>**<0.001**
Missing	113	27	86	
**Overall functional status (PCFS)**	2 (0, 3); Range: 0, 4	0 (0, 1); Range: 0, 4	3 (1, 3); Range: 0, 4	**<0.001**
Missing	53	10	43	
**Physical activity level (Frändin/Grimby)**	3 (2, 3); Range: 1, 6	3 (3, 4); Range: 1, 6	3 (2, 3); Range: 1, 6	**<0.001**
Missing	25	8	17	
**Depression (PHQ-9)**	9 (4, 14); Range: 0, 27	4 (2, 9); Range: 0, 23	9 (5, 14); Range: 0, 27	**<0.001**
Missing	96	15	81	
**Anxiety (GAD-7)**	4 (1, 9); Range: 0, 21	3 (0, 7); Range: 0, 18	5 (2, 10); Range: 0, 21	**<0.001**
Missing	88	14	74	

Data are presented as n (%); Mean (SD); Median (Q1, Q3); Range: Min, Max. Group differences were assessed using t-test or the Wilcoxon rank-sum test for continuous variables and Chi-squared or Fisher´s exact test for categorical variables depending on data level. P-values were corrected for multiple testing using the Benjamini & Hochberg correction for multiple testing, and adjusted p-values are reported.
Sensitivity analysis: Participants classified as Non-RSG despite clinically relevant dyspnoea (mMRC ≥2) were excluded (n = 39).
Abbreviations: EQ-VAS (0-100) - in the EuroQol Visual Analogue Scale; FEV_1 = Forced Expiratory Volume in 1 second; FEV_1/FVC = Ratio of FEV_1 to FVC; FVC = Forced Vital Capacity; GAD-7 = Generalized Anxiety Disorder-7 (0-21); mMRC = Modified Medical Research Council dyspnea scale (0-4); MIP = Maximal Inspiratory Pressure; PCFS = Post-COVID-19 Functional Status (0-4); PEF = Peak Expiratory Flow; PHQ-9 = Patient Health Questionnaire-9 (0-27); POTS = Postural Orthostatic Tachycardia Syndrome; 1MSTST = 1-Minute Sit-to-Stand Test; 6MWT = 6-Minute Walk Test; $p < 0.05$ considered statistically significant

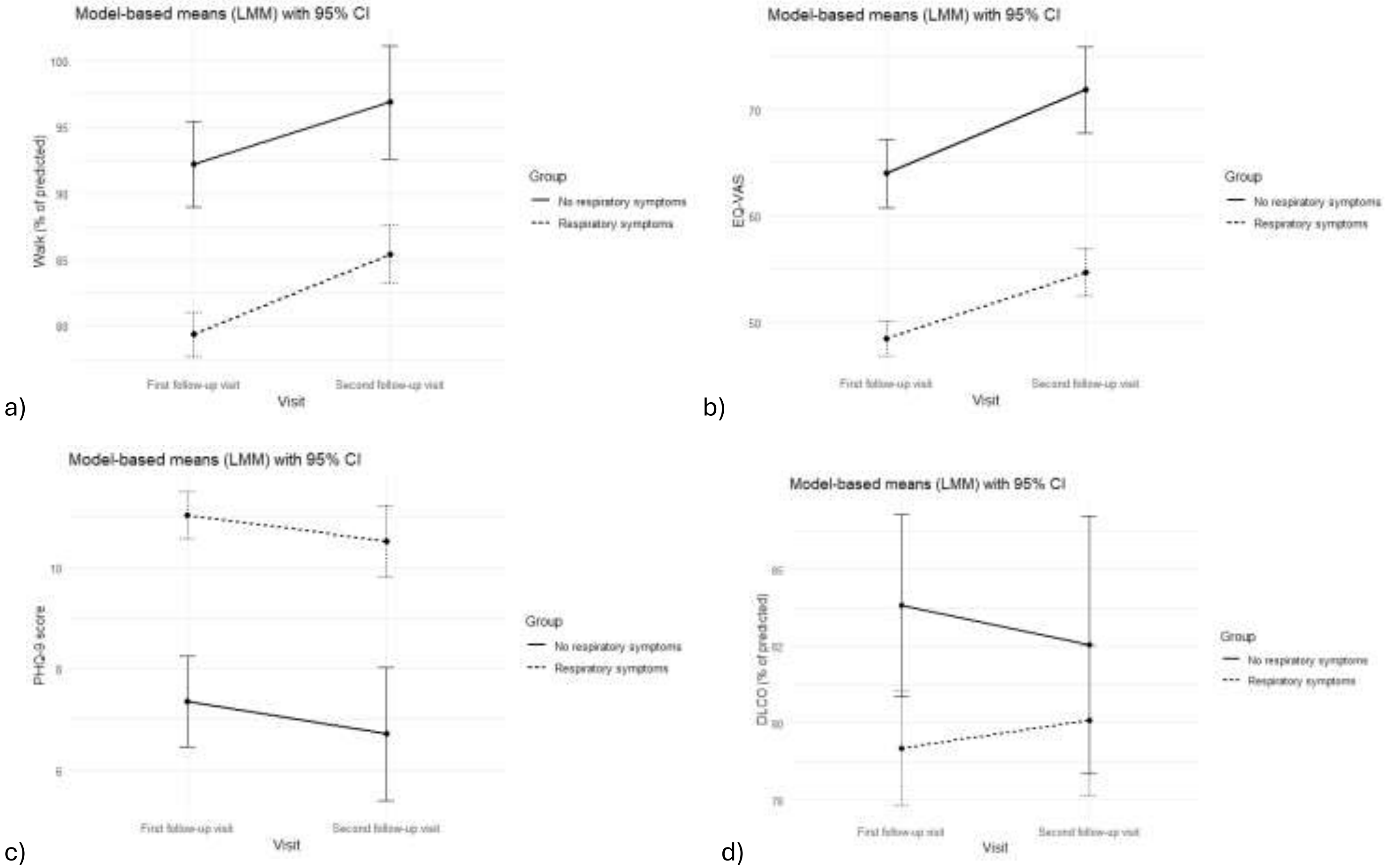

Fig. S1. Model-based estimated means (linear mixed models) with 95% confidence intervals for (a) 6-minute walk distance (% of predicted), (b) EQ-VAS score, (c) PHQ-9 score, and (d) DLCO (% of predicted) at first and second follow-up visits, stratified by groups (Non-respiratory symptom group vs respiratory symptom group) at first follow-up visit. Self-rated health was measured using the EQ-VAS (0–100) of the EuroQol Visual Analogue Scale. Depressive symptoms were assessed with the Patient Health Questionnaire-9 (PHQ-9; 0–27). DLCO indicates diffusing capacity for carbon monoxide.

Table S2. Results from linear mixed-effects models, presenting estimated marginal means at the first follow-up visit (T1) and the second follow-up visit (T2), within-group change (Δ), and the between-group interaction effect (ΔΔ) comparing the Non respiratory symptom group (non-RSG) and Respiratory symptom group (RSG).

Outcome	Group	N (T1/T2)	T1 EMM (95% CI)	T2 EMM (95% CI)	Δ (T2–T1) (95% CI for Δ)	p-value	ΔΔ (95% CI for ΔΔ)	p-value (interaction)
**MIP (% of predicted)**	No respiratory symptoms	203/119	95.00 (90.81, 99.19)	102.32 (96.74, 107.90)	7.32 (2.50, 12.14)	0.003	4.50 (-0.93, 9.93)	0.338
	Respiratory symptoms	760/441	87.12 (84.97, 89.27)	98.94 (96.03, 101.85)	11.82 (9.33, 14.32)	<0.001		
**1MSTST (% of predicted)**	No respiratory symptoms	203/119	72.39 (68.70, 76.09)	77.90 (72.89, 82.91)	5.51 (1.12, 9.89)	0.014	-1.64 (-6.63, 3.34)	0.748
	Respiratory symptoms	760/441	61.40 (59.46, 63.35)	65.27 (62.57, 67.97)	3.86 (1.48, 6.24)	0.002		
**6MWT (% of predicted)**	No respiratory symptoms	203/119	92.21 (88.95, 95.47)	96.90 (92.64, 101.16)	4.69 (1.17, 8.22)	0.009	1.35 (-2.63, 5.32)	0.748
	Respiratory symptoms	760/441	79.39 (77.71, 81.07)	85.43 (83.22, 87.64)	6.04 (4.21, 7.87)	<0.001		
**Self-rated health (EQ-VAS)**	No respiratory symptoms	203/119	64.03 (60.79, 67.27)	71.88 (67.79, 75.97)	7.85 (4.05, 11.65)	<0.001	-1.62 (-5.95, 2.70)	0.748
	Respiratory symptoms	760/441	48.50 (46.86, 50.14)	54.72 (52.50, 56.94)	6.22 (4.16, 8.29)	<0.001		
**Depression (PHQ-9)**	No respiratory symptoms	203/119	7.36 (6.46, 8.25)	6.72 (5.41, 8.03)	-0.64 (-1.88, 0.61)	0.313	0.12 (-1.29, 1.53)	0.864
	Respiratory symptoms	760/441	11.04 (10.57, 11.50)	10.52 (9.82, 11.22)	-0.52 (-1.18, 0.15)	0.259		

Anxiety (GAD-7)	No respiratory symptoms	203/119	5.41 (4.62, 6.20)	5.18 (4.06, 6.30)	-0.23 (-1.27, 0.81)	0.877	0.27 (-0.91, 1.46)	0.768
	Respiratory symptoms	760/441	7.30 (6.89, 7.72)	7.35 (6.74, 7.95)	0.04 (-0.52, 0.61)	0.877		
Physical Activity level (Frändin/Grimby)	No respiratory symptoms	203/119	3.39 (3.24, 3.53)	3.75 (3.53, 3.96)	0.36 (0.15, 0.57)	<0.001	0.06 (-0.18, 0.29)	0.768
	Respiratory symptoms	760/441	2.70 (2.63, 2.78)	3.12 (3.01, 3.23)	0.42 (0.31, 0.53)	<0.001		
Overall functional status (PCFS)	No respiratory symptoms	203/119	2.08 (1.91, 2.25)	1.86 (1.60, 2.12)	-0.22 (-0.47, 0.03)	0.087	0.05 (-0.24, 0.34)	0.793
	Respiratory symptoms	760/441	3.13 (3.04, 3.22)	2.96 (2.82, 3.10)	-0.17 (-0.31, -0.03)	0.030		
Total symptoms (No. of self-reported symptoms)	No respiratory symptoms	203/119	4.44 (3.71, 5.17)	2.72 (1.79, 3.65)	-1.72 (-2.77, -0.67)	0.001	-0.39 (-1.57, 0.79)	0.748
	Respiratory symptoms	760/441	8.06 (7.69, 8.44)	5.96 (5.47, 6.44)	-2.11 (-2.65, -1.56)	<0.001		
Dyspnoea severity (mMRC)	No respiratory symptoms	203/119	2.05 (1.89, 2.21)	1.97 (1.76, 2.19)	-0.08 (-0.29, 0.14)	0.486	-0.28 (-0.52, -0.03)	0.277
	Respiratory symptoms	760/441	3.10 (3.02, 3.18)	2.74 (2.63, 2.86)	-0.35 (-0.47, -0.23)	<0.001		
Self-perceived external dyspnoea	No respiratory symptoms	203/119	0.36 (0.15, 0.57)	0.61 (0.27, 0.95)	0.25 (-0.11, 0.61)	0.177	-0.38 (-0.79, 0.02)	0.277

**at rest (Borg CR10)**	Respiratory symptoms	760/441	1.41 (1.30, 1.52)	1.27 (1.10, 1.45)	-0.13 (-0.32, 0.05)	0.177		
**Self-perceived external dyspnoea after 6MWT (Borg CR10)**	No respiratory symptoms	203/119	3.11 (2.78, 3.45)	3.59 (3.07, 4.11)	0.48 (-0.05, 1.01)	0.152	-0.59 (-1.19, 0.00)	0.277
	Respiratory symptoms	760/441	4.84 (4.66, 5.01)	4.72 (4.45, 4.99)	-0.11 (-0.39, 0.16)	0.417		
**Self-perceived external dyspnoea after 1MSTST (Borg CR10)**	No respiratory symptoms	203/119	3.83 (3.50, 4.16)	3.91 (3.43, 4.39)	0.08 (-0.44, 0.60)	0.751	-0.20 (-0.78, 0.39)	0.748
	Respiratory symptoms	760/441	5.13 (4.97, 5.29)	5.02 (4.76, 5.27)	-0.11 (-0.39, 0.16)	0.751		

Values are estimated marginal means (95% CI) from linear mixed-effects models. T1/T2 = Follow-up 1/2; Δ = within-group change; ΔΔ = difference-in-difference (Respiratory symptoms vs No respiratory symptoms). P-values were corrected for multiple testing using the Benjamini & Hochberg correction for multiple testing, and adjusted p-values are reported.

Abbreviations: CI = Confidence Interval; EQ-VAS (0-100) - in the EuroQol Visual Analogue Scale; GAD-7 = Generalized Anxiety Disorder-7 (0-21); MIP = Maximal Inspiratory Pressure; mMRC = Modified Medical Research Council dyspnea scale (0-4); PCFS = Post-COVID-19 Functional Status (0-4); PHQ-9 = Patient Health Questionnaire-9 (0-27); POTS = Postural Orthostatic Tachycardia Syndrome; 1MSTST = 1-Minute Sit-to-Stand Test; 6MWT = 6-Minute Walk Test; $p < 0.05$ considered statistically significant

Table S3. Results from linear mixed-effects models showing estimated marginal means for extended pulmonary function tests at the first follow-up visit (T1) and the second follow-up visit (T2), including within-group change (Δ) and the between-group interaction effect (ΔΔ) comparing the Non-respiratory symptom group (non-RSG (n=27) and the Respiratory symptom group (RSG)(n=86).

Outcome	Group	N (T1/T2)	T1 EMM (95% CI)	T2 EMM (95% CI)	Δ (T2–T1) (95% CI for Δ)	p-value	ΔΔ (95% CI for ΔΔ)	p-value (interaction)
**FVC (% of predicted)**	No respiratory symptoms	27/27	84.67 (77.43, 91.90)	85.78 (78.55, 93.01)	1.11 (-2.22, 4.44)	0.510	1.94 (-1.86, 5.73)	0.551
	Respiratory symptoms	86/86	82.56 (78.61, 86.51)	85.60 (81.66, 89.55)	3.05 (1.23, 4.87)	0.001		
**FEV_1 (% of predicted)**	No respiratory symptoms	27/27	87.74 (80.75, 94.73)	88.37 (81.38, 95.36)	0.63 (-3.42, 4.68)	0.759	2.63 (-1.99, 7.24)	0.551
	Respiratory symptoms	86/86	82.55 (78.73, 86.37)	85.80 (81.98, 89.62)	3.26 (1.04, 5.47)	0.004		
**VC (% of predicted)**	No respiratory symptoms	27/27	83.59 (76.52, 90.67)	85.22 (78.15, 92.30)	1.63 (-1.03, 4.29)	0.228	1.71 (-1.33, 4.74)	0.551
	Respiratory symptoms	86/86	83.02 (79.16, 86.89)	86.36 (82.50, 90.22)	3.34 (1.88, 4.79)	<0.001		
**TLC (% of predicted)**	No respiratory symptoms	27/27	82.15 (75.25, 89.04)	84.56 (77.66, 91.45)	2.41 (-0.36, 5.18)	0.088	0.96 (-2.21, 4.13)	0.753
	Respiratory symptoms	84/84	86.30 (82.53, 90.07)	89.67 (85.90, 93.44)	3.37 (1.84, 4.90)	<0.001		
**FRC (% of predicted)**	No respiratory symptoms	27/27	92.44 (74.59, 110.30)	98.19 (80.33, 116.04)	5.74 (0.02, 11.46)	0.049	1.09 (-5.44, 7.63)	0.850
	Respiratory symptoms	84/84	98.15 (88.40, 107.91)	104.99 (95.23, 114.75)	6.83 (3.67, 9.99)	<0.001		
**RV (% of predicted)**	No respiratory symptoms	27/27	84.00 (70.54, 97.46)	88.56 (75.09, 102.02)	4.56 (-2.94, 12.05)	0.231	-0.81 (-9.37, 7.76)	0.850

	Respiratory symptoms	84/84	98.02 (90.66, 105.39)	101.77 (94.41, 109.14)	3.75 (-0.39, 7.89)	0.075		
**DLCO (% of predicted)**	No respiratory symptoms	25/25	79.32 (71.76, 86.88)	78.60 (71.04, 86.16)	-0.72 (-4.26, 2.82)	0.687	2.57 (-1.46, 6.59)	0.551
	Respiratory symptoms	84/84	72.57 (68.45, 76.70)	74.42 (70.29, 78.54)	1.85 (-0.08, 3.77)	0.061		

Values are estimated marginal means (95% CI) from linear mixed-effects models. T1/T2 = Follow-up 1/2; Δ = within-group change; ΔΔ = difference-in-difference (Respiratory symptoms vs No respiratory symptoms). P-values were corrected for multiple testing using the Benjamini & Hochberg correction for multiple testing, and adjusted p-values are reported. Abbreviations: CI = Confidence Interval; DLCO = Diffusing Capacity for Carbon Monoxide; FEV1 = Forced Expiratory Volume in 1 second; FEV1/FVC = Ratio of FEV1 to FVC; FRC = Functional Residual Capacity; FVC = Forced Vital Capacity; PFT = Pulmonary function test; RV = Residual Volume; TLC = Total Lung Capacity; VC = Vital Capacity

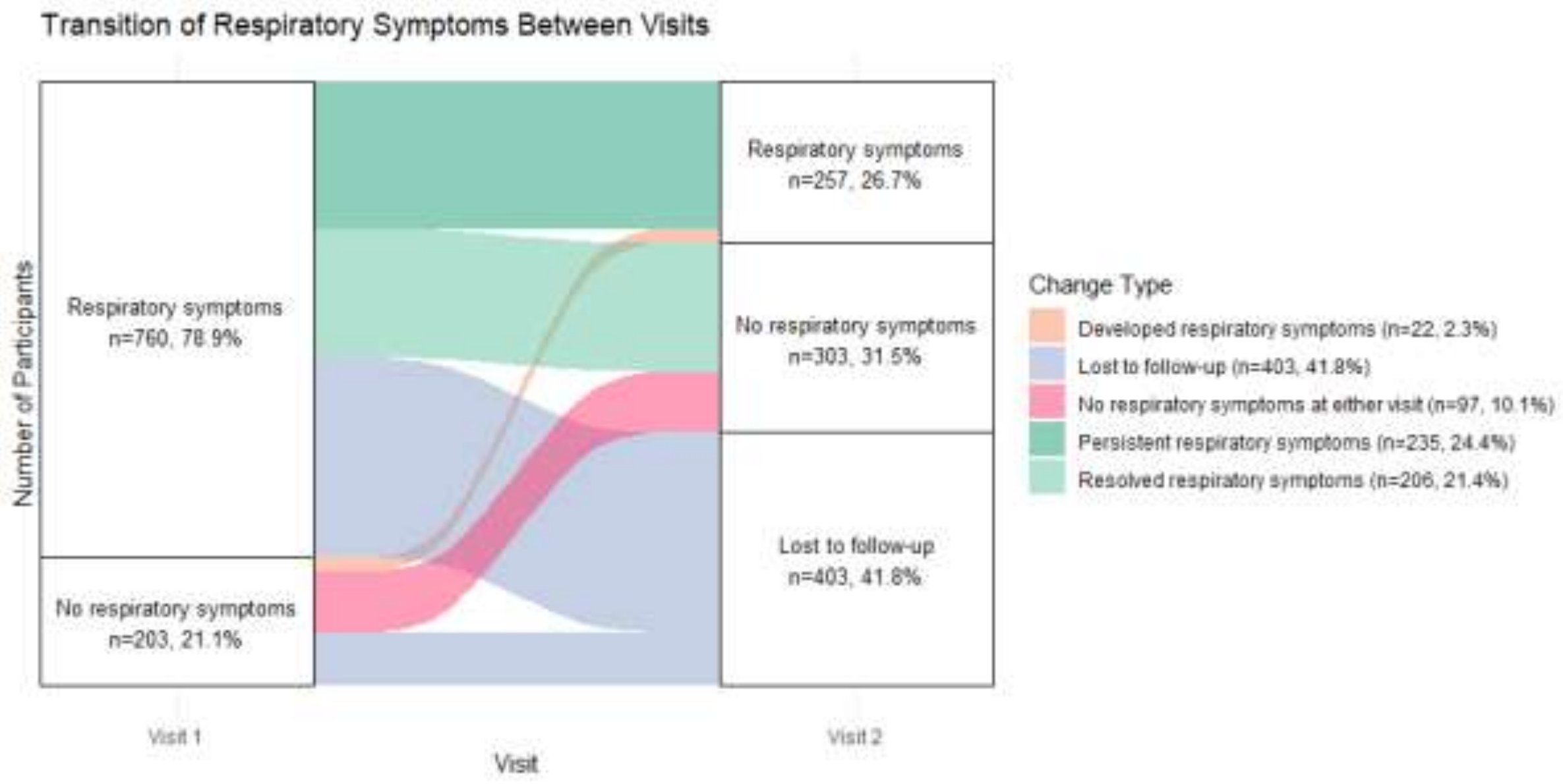

Fig. S2. Participant group transitions by respiratory symptom status between two visits.

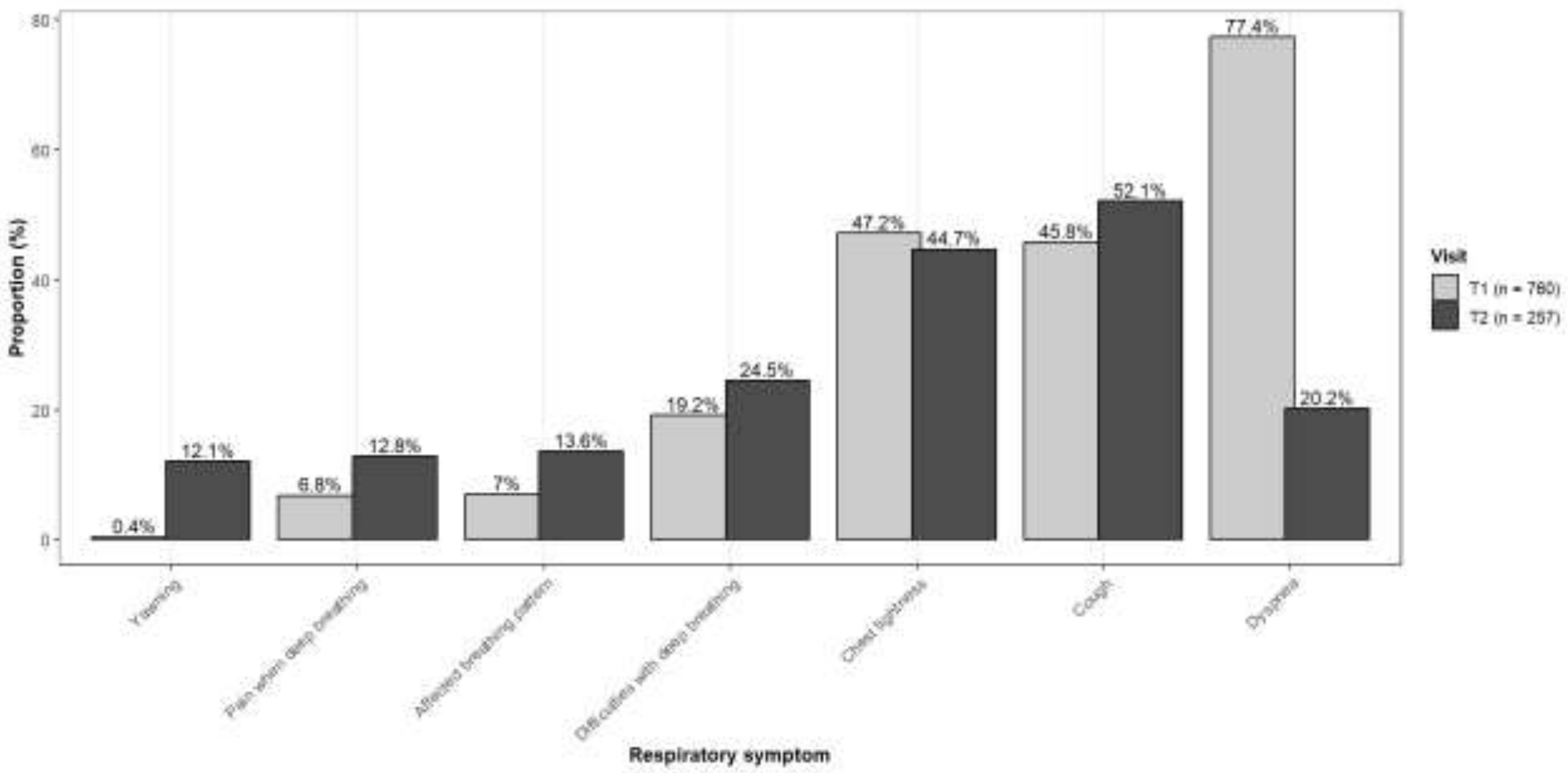

Fig. S3. Percentage distribution of specific respiratory symptoms in the respiratory symptom group (RSG) at first and second follow-up visit

Table S4. Sensitivity analysis excluding respiratory symptom status at the first follow-up visit: Univariable and multivariable logistic regression analyses (n=560) for predictors of belonging to the Respiratory symptom group (RSG) at the second follow-up visit (median 18 months post infection)

	**Univariat**					**Multivariat**			
	**n**	**OR**	**95% CI**	**p-value**	**p.adj (BH)**	**OR**	**95% CI**	**p-value**	**p.adj (BH)**
**First visit assessment**									
**6MWT (meter)**	545	1.00	1.00, 1.00	**<0.001**	**<0.01**	1.00	1.00, 1.00	**<0.05**	0.332
**MIP (cmH_2O)**	549	1.00	0.99, 1.00	0.061	0.086	1.00	0.99, 1.01	0.796	0.810
**1MSTST (No. of repetitions)**	541	0.97	0.95, 0.99	**<0.01**	**<0.01**	1.02	0.99, 1.05	0.274	0.810
**Self-rated health (EQ-VAS)**	504	0.99	0.98, 1.00	**<0.01**	**<0.05**	1.01	0.99, 1.02	0.396	0.810
Depression (GAD-7)	520	1.05	1.02, 1.08	**<0.01**	**<0.05**	1.01	0.95, 1.07	0.810	0.810
**Anxiety (PHQ-9)**	527	1.05	1.01, 1.08	**<0.01**	**<0.01**	0.99	0.94, 1.05	0.777	0.810
**Overall functional status (PCFS)**	538	1.24	1.09, 1.42	**<0.01**	**<0.01**	1.14	0.89, 1.45	0.305	0.810
**Physical Activity level (Frändin/Grimby)**	546	0.76	0.64, 0.90	**<0.01**	**<0.01**	1.15	0.85, 1.55	0.361	0.810
**Total symptoms** (No. of self-reported symptoms)	560	1.07	1.03, 1.11	**<0.001**	**<0.01**	1.05	0.98, 1.12	0.187	0.798
**FVC (L)**	523	0.76	0.63, 0.92	**<0.01**	**<0.05**	0.79	0.57, 1.07	0.134	0.759
**Clinically relevant dyspnoea (mMRC ≥ 2) (Yes)**	486	5.71	2.37, 17.0	**<0.001**	**<0.01**	6.10	2.07, 22.7	**<0.01**	**<0.05**
**Time from acute COVID-19 to first visit (Months)**	554	1.00	0.98, 1.03	0.734	0.832	1.01	0.96, 1.06	0.668	0.810
**Sex (F)**	560	1.47	1.05, 2.06	**<0.05**	**<0.05**	0.87	0.46, 1.65	0.677	0.810
**Age**	560	1.00	0.99, 1.01	0.948	0.976	1.00	0.97, 1.02	0.623	0.810
**POTS (Yes)**	560	1.01	0.62, 1.64	0.976	0.976	0.81	0.40, 1.63	0.551	0.810

	Univariat					Multivariat			
	n	OR	95% CI	p-value	p.adj (BH)	OR	95% CI	p-value	p.adj (BH)
**Hospitalized (Yes)**	560	0.89	0.64, 1.24	0.492	0.597	1.28	0.54, 3.04	0.573	0.810
**Asthma (Yes)**	555	1.24	0.79, 1.93	0.4	0.5	0.96	0.49, 1.66	0.743	0.810

P-values were corrected for multiple testing using the Benjamini & Hochberg correction for multiple testing, and adjusted p-values are reported, Time to second follow-up visit, median 18 months. Abbreviations: CI = Confidence Interval; GAD-7 = measured with Generalized Anxiety Disorder-7 (0-21); mMRC = Modified Medical Research Council dyspnea scale using ≥ 2 as a cut off; MIP = Maximal Inspiratory Pressure; OR = Odds ratio; PCFS = Post-COVID-19 Functional Status (0-4); PHQ-9 = Patient Health Questionnaire-9 (0-27); POTS = Postural Orthostatic Tachycardia Syndrome; EQ-VAS (0-100)- in the EuroQol Visual Analogue Scale; 1MSTST = 1-Minute Sit-to-Stand Test; 6MWT = 6-Minute Walk Test; $p < 0.05$ considered statistically significant
